# A National Analysis of Alcohol Withdrawal Syndrome in Patients with Operative Trauma

**DOI:** 10.1016/j.sopen.2024.05.001

**Published:** 2024-05-12

**Authors:** Jeffrey Balian, Nam Yong Cho, Amulya Vadlakonda, Joanna Curry, Nikhil Chervu, Konmal Ali, Peyman Benharash

**Affiliations:** Department of Surgery, David Geffen School of Medicine, University of California, Los Angeles, Los Angeles, CA, USA

**Keywords:** Trauma surgery, Alcohol withdrawal syndrome

## Abstract

**Background:**

Alcohol withdrawal syndrome (AWS) presents with a complex spectrum of clinical manifestations that complicate postoperative management. In trauma setting, subjective screening for AWS remains challenging due to the criticality of injury in these patients. We thus identified several patient characteristics and perioperative outcomes associated AWS development.

**Methods:**

The 2016–2020 National Inpatient Sample was queried to identify all non-elective adult (≥18 years) hospitalizations for blunt or penetrating trauma undergoing operative management with a diagnosis of AWS. Patients with traumatic brain injury or with a hospital duration of stay <2 days were excluded. Outcomes of interest included in-hospital mortality, perioperative complications, hospitalization costs, length of stay (LOS) and non-home discharge.

**Results:**

Of an estimated 2,965,079 operative trauma hospitalizations included for analysis, 36,415 (1.23 %) developed AWS following admission. The AWS cohort demonstrated increased odds of mortality (Adjusted Odds Ratio [AOR] 1.46, 95 % Confidence Interval [95 % CI] 1.23–1.73), along with infectious (AOR 1.73, 95 % CI 1.58–1.88), cardiac (AOR 1.24, 95 % CI 1.06–1.46), and respiratory (AOR 1.96, 95 % CI 1.81–2.11) complications. AWS was associated with prolonged LOS, (β: 3.3 days, 95 % CI: 3.0 to 3.5), greater cost (β: +$8900, 95 % CI $7900–9800) and incremental odds of nonhome discharge (AOR 1.43, 95 % CI 1.34–1.53). Furthermore, male sex, Medicaid insurance status, head injury and thoracic operation were linked with greater odds of development of AWS.

**Conclusion:**

In the present study, AWS development was associated with increased odds of in-hospital mortality, perioperative complications, and resource burden. The identification of patient and operative characteristics linked with AWS may improve screening protocols in trauma care.

## Introduction

In the United States, alcohol withdrawal syndrome (AWS) imposes a significant burden on the healthcare system, with nearly 500,000 hospitalized patients impacted annually [1]. Often triggered by sudden reduction or cessation of alcohol consumption, AWS presents with a complex spectrum of manifestations including severe cognitive derangements [2]. The severity of the syndrome is evident as nearly 15 % of affected individuals progress to seizures, while up to 5 % are at risk of delirium tremens [3]. Early detection and appropriate treatment remain the cornerstones of the management of AWS. Nevertheless, among those with traumatic injuries, inadvertent periods of abstinence following critical illness and surgical procedures pose a challenge in the screening process.

Prior work has noted nearly half of all admissions at US trauma centers to exhibit measurable blood alcohol levels, with 1–2 % of patients subsequently manifesting AWS [4]. In response, some institutions have implemented screening practices, such as the Clinical Institute Withdrawal Assessment for Alcohol (CIWA) protocol, aimed at mitigating the development of AWS among inpatients [5]. However, the applicability of such subjective assessments is limited in trauma patients due to their high acuity of injury or altered mental status [6]. Notably, consortium-based investigators have noted AWS patients progressing to delirium tremens to face a substantial increase in-hospital mortality [7]. Additionally, previous studies have noted the higher odds of needing mechanical ventilation and heightened susceptibility to respiratory infections among AWS patients, often resulting in a prolonged duration of stay [4].

However, available literature examining AWS among hospitalized trauma victims predominantly focuses on the nonoperative cohort [[8], [9], [10]]. With implications towards perioperative management and resource utilization, we utilized a national cohort to characterize clinical and financial outcomes in patients with AWS following operations for traumatic injury. We hypothesized AWS to be independently associated with increased odds of in-hospital mortality, postoperative complications, and resource utilization. Additionally, we sought to determine patient and hospital characteristics associated with AWS which may inform novel risk stratification metrics.

## Methods

**Fig. 1:**
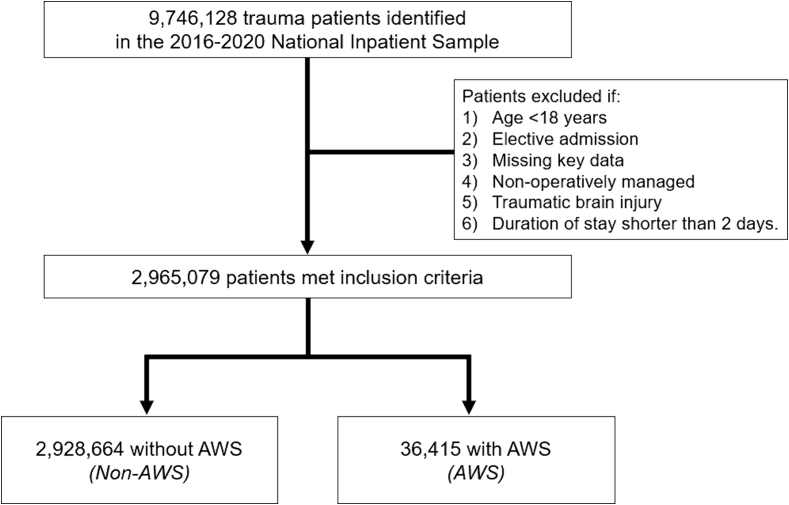
Consort (Consolidated Standards of Reporting Trials) diagram of study cohort and survey-weighted sample size. *AWS*, alcohol withdrawal syndrome.

This was a retrospective study of the 2016–2020 National Inpatient Sample (NIS). The NIS is the largest all-payer discharge database and uses validated sampling algorithms to provide national estimates for ~97 % of all US hospitalizations [11]. All non-elective adult (≥18 years) hospitalizations for blunt or penetrating trauma undergoing operative management were included for analysis. Relevant *International Classification of Diseases*, *Tenth Revision* (ICD-10) diagnosis codes were used to identify patients who developed alcohol withdrawal syndrome following admission (Supplementary Table 1). The cohort was subsequently grouped into those with AWS and no AWS (Non-AWS). To reduce heterogeneity, patients with traumatic brain injury or with a hospital duration of stay <2 days were excluded from the analysis. Additionally, records with missing data for age, sex and hospitalization costs, were excluded (<1 % of those missing variables) (Fig. 1).

The NIS data dictionary was used to define patient and hospital characteristics, including age, sex, race, income quartile, insurance status, hospital geographic region, teaching status, and bed size. Patients identified as having a psychiatric disorder included those with a diagnosis of depression, psychosis and non-substance-related cognitive disorders. Opioid use disorder was identified using a previously defined methodology [12]. Injury severity was measured using the ICD-derived Trauma Mortality Prediction Model (TMPM), which generates the likelihood of mortality (range: 0–100) based on available ICD codes [13]. Using the methods of Dobaria et al., operations were classified as facial, neck, thoracic, cardiac, gastrointestinal, genitourinary, vascular and orthopedic/plastic [14]. Injury location was stratified into head/neck, face, chest, abdomen/pelvis and upper/lower extremity regions (Supplementary Table 2). To adjust for patients with multiple injuries, location, operation and mechanism were transformed from discrete to binary classification.

The primary outcome of interest was in-hospital mortality, while secondary outcomes included perioperative complications, length of stay (LOS), hospitalization costs and non-home discharge. Complications were categorized into cardiac (cardiac arrest, ventricular tachycardia, ventricular fibrillation, cardiac tamponade, myocardial infarction), respiratory (pneumothorax, pneumonia, acute respiratory failure, prolonged ventilation), neurologic (transient ischemic attack and cerebral infarction), renal (acute kidney injury), infectious (sepsis, septicemia, bacteremia, and central line bloodstream infection), thromboembolic (deep vein thrombosis, pulmonary embolism), and intraoperative (accidental puncture). Non-home discharge was defined as disposition to a short-term hospital, skilled nursing facility, or intermediate care facility. Hospitalization costs were calculated by applying center-specific cost-to-charge ratios to overall charges, followed by inflation adjustment to the 2020 Personal Healthcare Index [15].

Categorical variables are reported as proportions (%) while continuous variables are described as medians with interquartile range (IQR). Pearson's Chi-square and adjusted Wald tests were used to compare categorical and continuous variables, respectively. Cuzick's non-parametric test (nptrend) was utilized to evaluate the significance of temporal trends [16]. Covariate selection was performed using elastic net regularization, which minimizes overfitting and collinearity via a penalized least-squares methodology [17]. Linear and logistic multivariable regression models were developed to assess the independent association of AWS with outcomes of interest. Regression outcomes are reported as adjusted odds ratio (AOR) and β coefficients, as appropriate, with 95 % confidence intervals (95 % CI). Statistical significance was with α at 0.05. All statistical analysis was performed using Stata software version 16.1 (StataCorp, College Station, TX). This study was deemed exempt from full review by the Institutional Review Board at the University of California, Los Angeles.

## Results

### Baseline demographics and outcomes

Of an estimated 2,965,079 operative trauma patients included for analysis, 36,415 (1.23 %) developed AWS following admission for trauma. The incidence of AWS remained steady over the study period (2016: 1.2 % vs 2020: 1.2 %, nptrend = 0.32). Patients with AWS were younger (59 [50–67] vs 72 [55–83] years, *P* < 0.001) and less commonly female (26.1 vs 57.1 %, *P* < 0.001) compared to others. (Table 1). Compared to others, AWS patients were more commonly in the lowest income quartile (32.0 vs 28.6 %, *P* < 0.001), insured by Medicaid (27.0 vs 10.4 %, P < 0.001) and treated at non-teaching hospitals (20.0 vs 19.0 %, *P* < 0.05). Moreover, the AWS cohort more frequently had coagulopathy (23.5 vs 7.0 %), liver disease (19.7 vs 2.8 %) and psychiatric disorders (16.8 vs 13.7 %) (all *P* < 0.001). Patients with AWS had increased severity of injury measured by TMPM (0.8 [0.6–6.0] vs 0.8 [0.5–1.0], *P* < 0.001) compared to those without. Those with AWS had a greater proportion of head/neck (23.6 vs 13.0 %, *P* < 0.001) and fall-related (77.9 vs 75.4 %, *P* = 0.01) injuries compared to non-AWS (Table 2). Finally, patients developing AWS had a greater incidence of facial (3.4 vs 1.5, *P* < 0.001), thoracic (3.6 vs 1.4, P < 0.001), and gastrointestinal surgeries (6.6 vs 4.2, P < 0.001) compared to others.

**Table 1:** Demographic and clinical characteristics of AWS vs non-AWS after trauma operation.

	Non-AWS (*n* = 2,928,664)	AWS (*n* = 36,415)	*P*-value
Age (y), median [IQR]	72 [55–83]	59 [50–67]	<0.001
Female (%)	57.1	26.1	<0.001
Trauma mortality prediction score (%)	0.8 [0.5–1.0]	0.8 [0.6–6.0]	<0.001
Race (%)			<0.001
White	76.6	76.8	
Black	9.6	9.9	
Hispanic	8.8	8.0	
Other race	4.9	5.1	
Primary insurance (%)			<0.001
Private	18.4	19.8	
Medicare	61.2	39.9	
Medicaid	10.4	27.0	
Other payers^a^	9.9	14.2	
Hospital setting			0.001
Metropolitan teaching	72.2	74.2	
Metropolitan non-teaching	20.0	19.0	
Non-Metropolitan	7.8	6.9	
Income quartile (%)			<0.001
76th–100th	20.5	18.5	
51st–75th	24.3	23.5	
26th–50th	26.6	26.1	
0–25th	28.6	32.0	
Comorbidities (%)			
Congestive heart failure	13.1	10.5	<0.001
Chronic liver disease	2.78	19.7	<0.001
Chronic lung disease	16.6	20.4	<0.001
Coagulopathy	7.03	23.5	<0.001
Electrolyte disorder	28.1	64.7	<0.001
Hypertension	55.6	46.8	<0.001
Psychiatric disorder	13.7	16.8	<0.001
Opioid use disorder	0.8	2.6	<0.001
Tobacco use disorder	15.7	36.8	<0.001
Cannabis use disorder	2.2	5.0	0.01

a Includes self-pay, uninsured, and other.

**Table 2:** Patient injury and operative characteristics of AWS vs non-AWS after trauma operation.

	Non-AWS (n = 2,928,664)	AWS (n = 36,415)	P-value
Multiple operation (%)	5.0	0.9	<0.001
Mechanism of injury (%)			
Fall	75.4	77.9	0.01
Gunshot wound	2.9	1.7	<0.001
Blunt injury	4.2	3.6	<0.001
Motor vehicle collision	7.6	6.8	0.03
Motorcycle collision	3.5	2.1	<0.001
Motor vehicle versus pedestrian	2.6	4.2	<0.001
Stab injury	3.1	4.3	<0.001
Location of injury (%)			
Head/neck	13.0	23.6	<0.001
Chest	10.0	15.5	<0.001
Abdomen	11.7	13.5	<0.001
Upper extremity	23.7	25.8	<0.001
Lower extremity	70.2	56.2	<0.001
Primary operation type (%)			
Orthopedic/plastic	91.4	84.0	<0.001
Facial	1.5	3.4	<0.001
Neck	0.7	2.2	<0.001
Thoracic	1.4	3.6	<0.001
Cardiac	1.9	2.2	0.03
Gastrointestinal	4.2	6.6	<0.001
Genitourinary	1.3	1.2	0.44
Vascular	1.9	2.8	<0.001

### Factors associated with development of alcohol withdrawal syndrome

Factors independently associated with AWS are shown in Table 3. Male sex, White race and admission to a rural hospital were associated with greater odds of AWS. Additionally, increasing age (per year) and Medicare insurance status were associated with lower odds of developing AWS. Chronic liver disease, coagulopathy, and psychiatric disorders were linked with increased odds of AWS. Injury to the head and thorax along with thoracic operation were linked with greater odds of AWS manifestation.

**Table 3:** Factors Associated with Development of AWS Among Patients Undergoing Trauma Operation (income quartile, 0–25th; insurance payer, Private; hospital setting, metropolitan teaching; race, white.)

	AOR	95 % CI	P-value
Age	0.98	0.98–0.98	<0.001
Female	0.31	0.30–0.33	<0.001
Race			<0.001
White	Reference		
Black	0.71	0.64–0.78	<0.001
Hispanic	0.62	0.56–0.68	<0.001
Other race	0.82	0.72–0.94	<0.001
Primary insurance			
Private	Reference		
Medicare	0.71	0.65–0.78	<0.001
Medicaid	1.87	1.73–2.02	<0.001
Other payer^a^	1.33	1.21–1.46	<0.001
Income quartile			
76th–100th	Reference		
51st–75th	0.99	0.91–1.07	0.72
26th–50th	0.97	0.90–1.06	0.52
0–25th	1.00	0.93–1.10	0.93
Hospital setting			
Metropolitan teaching	Reference		
Metropolitan non-teaching	1.12	1.04–1.20	<0.001
Non-Metropolitan	1.08	1.00–1.16	0.04
Hospital ownership			
Government hospital	Reference		
Private non-profit	0.98	0.91–1.06	0.70
Private for-profit	0.99	0.89–1.10	0.82
Primary operation type			
Orthopedic/plastic	0.93	0.84–1.03	0.15
Facial	1.11	0.94–1.32	0.22
Neck	1.48	1.23–1.78	<0.001
Thoracic	1.49	1.28–1.73	<0.001
Cardiac	0.43	0.36–0.52	<0.001
Gastrointestinal	1.12	0.98–1.27	0.09
Genitourinary	0.69	0.55–0.87	0.02
Vascular	0.87	0.73–1.01	0.08
Multioperation	0.94	0.80–1.08	0.38
Mechanism of injury			
Fall	1.95	1.61–2.37	<0.001
Gunshot wound	0.44	0.33–0.58	<0.001
Blunt injury	1.10	0.90–1.33	0.35
Motor vehicle collision	0.44	0.38–0.52	<0.001
Motorcycle collision	0.32	0.26–0.39	<0.001
Motor vehicle versus pedestrian	1.04	0.91–1.19	0.61
Stab Injury	1.01	0.81–1.24	0.98
Location of injury			
Head/neck	1.71	1.59–1.82	<0.001
Thorax	1.49	1.28–1.51	<0.001
Abdomen	1.00	0.92–1.09	0.96
Upper extremity	1.09	1.02–1.16	0.01
Lower extremity	0.93	0.88–0.99	0.03
Comorbidities			
Liver disease	2.57	2.39–2.76	<0.001
Psychiatric disorder	1.47	1.38–1.57	<0.001
Cardiac arrhythmia	1.14	1.06–1.23	<0.001
Coagulopathy	2.34	2.19–2.53	<0.001
Neurologic disorder	2.64	2.48–2.80	<0.001

### Outcome analysis

As shown in Table 4, patients manifesting AWS exhibited higher in-hospital mortality (2.1 vs 1.4 %, *P* < 0.001) compared to non-AWS. Of note, those with AWS had higher rates of cardiac (2.8 vs 1.7 %, *P* < 0.001) and respiratory (13.5 vs 5.6 %, P < 0.001) complications. Additionally, those with AWS demonstrated an increment in median hospital duration (8 [5–14] vs 5 [3–7] days, P < 0.001) and index hospitalization costs ($27,000 [$18,000–46,000] vs $18,000 [$13,000–27,000], P < 0.001). Over the study period, total healthcare cost attributable to AWS has significantly increased from $274 million in 2016 to $321 million in 2020 (nptrend = 0.03).

**Table 4:** Unadjusted and adjusted outcomes AWS compared to non-AWS. Categorical variables are reported with proportions (%) and Adjusted Odds Ratio (AOR) with 95 % confidence intervals (95 % CI). Continuous variables are reported using median with interquartile range and β coefficient with 95 % CI.

	Non-AWS	AWS	*P*-value
Clinical outcomes			
In-hospital mortality	1.4	2.1	<0.001
Infectious complications	3.8	9.7	<0.001
Intraoperative complications	0.3	0.4	0.03
Respiratory complications	5.6	13.5	<0.001
Cardiac complications	1.7	2.8	<0.001
Acute kidney injury	13.5	15.4	<0.001
Perioperative stroke	0.5	0.6	0.125
Non-home discharge	69.4	64.5	<0.001
Blood transfusion	13.1	15.1	<0.001
Resource utilization			
Length of stay (days)	5 [3–7]	8 [5–14]	<0.001
Cost ($1000)	18 [13–27]	27 [18–46]	<0.001

**Fig. 2:**
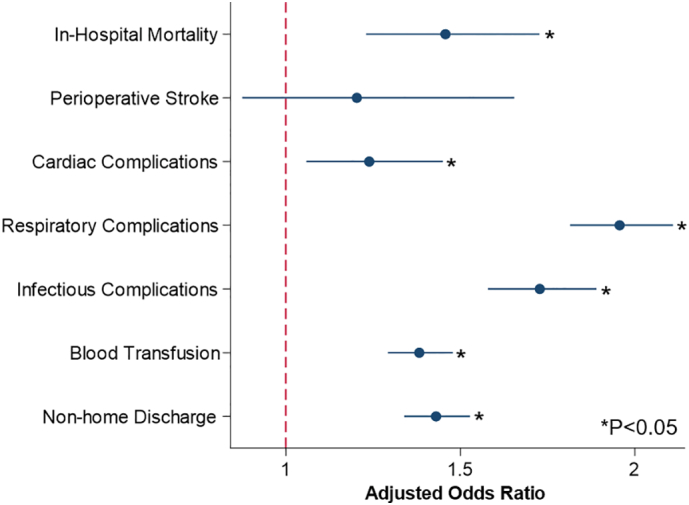
Adjusted outcomes of AWS following Trauma operation. Multivariable regression was created with reference condition represented by the red bar. The odds ratio is indicated by the dots, and the 95 % CIs are represented by horizontal blue lines. (For interpretation of the references to color in this figure legend, the reader is referred to the web version of this article.)

Following risk adjustment, AWS was associated with greater odds of mortality (AOR 1.46, 95 % CI 1.23–1.73) (Fig. 2). Development of AWS was also linked with greater odds of infectious (AOR 1.73, 95 % CI 1.58–1.88), cardiac (AOR 1.24, 95 % CI 1.06–1.45), and respiratory (AOR 1.96, 95 % CI 1.81–2.11) complications. Furthermore, AWS was associated with prolonged duration of stay by 3.3 days (95 % CI 3.0–3.5 days), greater hospitalization costs by $8900 (95 % CI $7900–9800) and incremental odds of non-home discharge (AOR 1.43, 95 % CI 1.34–1.53).

## Discussion

In the context of trauma care, development of AWS portends a notable risk for inpatient postoperative complications and increased resource utilization. The present study used a nationally representative cohort to examine the association of AWS with clinical and financial outcomes after operative traumatic injuries. We found younger age, White race, chronic liver disease, and psychiatric disorders to be associated with increased odds of developing AWS. Additionally, those with AWS incurred higher costs, prolonged LOS and demonstrated increased odds of mortality and perioperative complications. Several of these findings warrant further discussion.

The present study identified several patient characteristics, including liver disease, coagulopathy, and psychiatric disorder, to be significantly associated with the development of AWS following operation for traumatic injuries. Congruent with these findings, Ahmed et al. identified patients with AWS to have increased frequency of cirrhosis and coagulopathic disorder [18]. Additionally, in a single center cohort of ICU admissions, Vigouroux and colleagues reported 34.8 % AWS patients have comorbid psychiatric disorders [19]. In present day practice, the assessment of AWS severity is on the CIWA scale, which evaluates patient presentations such as anxiety, agitation, and headache to guide medication therapy. However, application of CIWA in critically ill patients remains limited due to the subjective nature and the requirement of patient participation. Our findings suggest increased vigilance and management of comorbid risk factors through screening strategies to enhance early AWS detection. Future assessment metrics accounting for objective and holistic parameters including abbreviated injury score, comorbid conditions and socioeconomic status may allow timely intervention in high-risk AWS patients in trauma settings.

Our findings demonstrated the manifestation of AWS to have increased odds of in-hospital mortality and greater severity of injury quantified by TMPM compared to non-AWS [13]. Furthermore, AWS patients showed a greater risk of postoperative cardiac, pulmonary and infectious complications in our analysis. Those with chronic alcohol dependence are predisposed to sympathetic hyperactivity, which heightens the risk of cardiac derangements. In particular, Kähkönen and colleagues reported acute risk of hypotension and arrhythmia in patients who develop AWS [20]. Consequently, managing withdrawal becomes crucial in mitigating hemodynamic disturbance exacerbation in those with traumatic injuries. Moreover, previous work has noted a 3-fold increase in ARDS and pneumonia as well as a >2-fold increase in sepsis among AWS patients [18]. Our findings corroborate the greater risks of respiratory and infectious complications in AWS patients undergoing operation for trauma. Additional studies delineating the effects of these treatment modalities on outcomes of trauma-related operations are warranted. Given the high perioperative risks of patients who develop AWS, multidisciplinary team efforts to optimize perioperative conditions may improve outcomes in operative trauma cases.

The financial burden attributed to AWS in trauma patients appears to have increased from $274 to 321 million over a 5-year period. Additionally, we found AWS to incur an incremental cost of nearly $8900 following operative trauma. While these findings may partly stem from the increased rates of surgical complications in this cohort, the financial burden may also be attributed to the management of AWS. Prior work has implicated AWS-associated treatment and sequelae as significant drivers of the cost in trauma hospitalization, noting increased needs for medical therapy and intubation [22]. Furthermore, increased resistance to sedatives, inadequate pain control and the development of concurrent AWS-related delirium or seizures may further contribute to these discrepancies in hospitalization costs [23]. Following risk adjustment, we also noted that AWS development had >3 days of incremental hospital duration of stay. Gupta et al. has also found AWS to be associated with prolonged LOS, noting increased transfer to the ICU, need for invasive mechanical ventilation and vasopressor use [24]. Additionally, prior work has noted delirium tremens is independently associated with 2.4-fold increase in intensive care unit admission and doubled duration of stay [25]. These findings underscore the criticality of early identification and screening, which may alleviate the resource burden imposed by AWS in trauma care.

The present study has several important limitations, including those inherent to using an administrative database and its retrospective nature. The use of ICD-based coding in the NIS may be influenced by the variation in regional and institutional billing practices. The absence of clinical granularity including vital signs, lab values, and chronicity of the alcohol consumption history could not be ascertained. Additionally, we were unable to adjust for operative characteristics such as complexity and time to operation, as well as surgeon experience. Furthermore, our clinical endpoints were limited to the duration of admission, which lacks insights into long-term outcomes such as out-of-hospital mortality, readmissions, and reoperations. Despite these limitations, our study used statistically validated methodologies and the largest available inpatient dataset to examine nationally representative outcomes.

While the prevalence of AWS following operations for traumatic injuries remained consistent over the study period, its related cost burden on healthcare has significantly increased. Moreover, risks of mortality and perioperative complications associated with AWS continue to pose risks in trauma patients undergoing an operation. While interventions to treat AWS in patients prevail, optimization of perioperative screening and conditioning is warranted in the setting of trauma. The identification of patient and hospital features associated with withdrawal may guide novel risk assessment tools. Multidisciplinary team efforts to identify and provide timely care for AWS may improve patient outcomes and resource allocation in trauma care. Policies to improve patient screening and detection with delayed operation may optimize perioperative conditions and improve outcomes in select trauma cases.

## Funding sources statement

The authors have no funding or financial support to report.

## Ethics approval statement

The data that support the findings of this study are available from the Healthcare Cost and Utilization Project (HCUP). Restrictions apply to the availability of these data, which were used with permission for this study. Data are available from the authors with the permission of the Healthcare Cost and Utilization Project. This study was deemed exempt from full review by the Institutional Review Board at the University of California, Los Angeles, due to the de-identified nature of the data.

## CRediT authorship contribution statement

**Jeffrey Balian:** Conceptualization, Data curation, Methodology, Writing – original draft. **Nam Yong Cho:** Methodology, Validation, Writing – review & editing. **Amulya Vadlakonda:** Validation, Writing – review & editing. **Joanna Curry:** Conceptualization, Writing – review & editing. **Nikhil Chervu:** Conceptualization, Validation. **Konmal Ali:** Writing – review & editing. **Peyman Benharash:** Conceptualization, Methodology, Supervision, Writing – review & editing.

## Declaration of competing interest

The authors have no related conflicts of interest or disclosures to report.

## Appendix A Supplementary data

Supplementary tables Image 1
