# Supplementary material for: A National Analysis of Alcohol Withdrawal Syndrome in Patients with Operative Trauma
Source: Surg Open Sci. 2024 May 12;19:199–204. doi: 10.1016/j.sopen.2024.05.001 (PMC11127230; doi:10.1016/j.sopen.2024.05.001)
Supplement: Supplementary file 1 — Supplementary tables [file mmc1.docx]

**Supplementary Table 1:** Administrative codes used to identify patients in with Alcohol Withdrawal Syndrome in the NIS.

|  | ICD-10 |
| --- | --- |
| Alcohol Withdrawal Syndrome | F12910, F12913, F129181, F10130, F10131, F10139, F10230, F10231, F10239, F10930, F10931, F10932, F10939 |

**Supplementary Table 2:** International Classification of Diseases 10^th^ Revision (ICD-10) codes for injury location.

| **Location of Injury** | **ICD-10 Codes** |
| --- | --- |
| Head | S00x, S03x, S04x, S05x, S08x, S10x, S11x, S12x, S13x, S14x, S15x, S16x, S17x, S18x, S19x |
| Thorax | S20x, S21x, S22x, S23x, S25x, S26x, S27x, S28x, S29x |
| Abdomen | S30x, S31x, S33x, S34x, S35x, S36x, S37x, S38x, S39x |
| Upper Extremity | S40x, S41x, S42x, S43x, S44x, S45x, S46x, S47x, S48x, S49x, S50x, S51x, S52x, S53x, S54x, S55x, S56x, S57x, S58x, S59x, S60, S61, S62x, S63x, S64x, S65x, S66x, S67x, S68x, S69x |
| Lower Extremity | S70x, S71x, S72x, S73x, S74x, S75x, S76x, S77x, S78x, S79x, S80x, S81x, S82x, S83x, S84x, S85x, S86x, S87x, S88x, S89x, S90x, S91x, S92x, S93x, S94x, S95x, S96x, S97x, S98x, S99x |

|  | ***Non-AWS*** | ***AWS*** | ***P-Value*** |
| --- | --- | --- | --- |
| **Clinical outcomes** |  |  |  |
| In-hospital mortality | 1.0 | 1.3 | 0.04 |
| Infectious complications | 2.7 | 6.9 | <0.001 |
| Intraoperative complications | 0.2 | 0.2 | 0.39 |
| Respiratory complications | 4.0 | 9.2 | <0.001 |
| Cardiac complications | 1.1 | 1.7 | <0.001 |
| Acute Kidney Injury | 12.5 | 13.3 | 0.06 |
| Perioperative Stroke | 0.4 | 0.4 | 0.27 |
| Non-home discharge | 72.9 | 68.0 | <0.001 |
| Blood transfusion | 12.7 | 14.8 | <0.001 |
| **Resource utilization** |  |  |  |
| Length of stay (days) | 5 [3-7] | 8 [5-14] | <0.001 |
| Cost ($1,000) | 18 [18-25] | 25 [17-39] | <0.001 |

**Supplementary Table 3A**: Of patients with orthopedic or plastic operations, unadjusted outcomes are compared between AWS and non-AWS. Categorial and continuous variables are reported with proportions (%) and median with interquartile range, respectively.

|  | ***Non-AWS*** | ***AWS*** | ***P-Value*** |
| --- | --- | --- | --- |
| **Clinical outcomes** |  |  |  |
| In-hospital mortality | 0.5 | 0.9 | 0.44 |
| Infectious complications | 2.0 | 6.2 | <0.001 |
| Intraoperative complications | 0.2 | 0.9 | 0.04 |
| Respiratory complications | 5.5 | 15.4 | <0.001 |
| Cardiac complications | 0.8 | 2.2 | 0.02 |
| Acute Kidney Injury | 5.5 | 9.7 | 0.01 |
| Perioperative Stroke | 0.2 | 0 | 0.48 |
| Non-home discharge | 15.8 | 30.0 | <0.001 |
| Blood transfusion | 3.8 | 9.7 | <0.001 |
| **Resource utilization** |  |  |  |
| Length of stay (days) | 3 [2-6] | 6 [4-10] | <0.001 |
| Cost ($1,000) | 17 [12-27] | 23 [15-36] | <0.001 |

**Supplementary Table 3B**: Of patients with facial operations, unadjusted outcomes are compared between AWS and non-AWS. Categorial and continuous variables are reported with proportions (%) and median with interquartile range, respectively.

|  | ***Non-AWS*** | ***AWS*** | ***P-Value*** |
| --- | --- | --- | --- |
| **Clinical outcomes** |  |  |  |
| In-hospital mortality | 5.8 | 3.3 | 0.25 |
| Infectious complications | 22.2 | 32.0 | 0.01 |
| Intraoperative complications | 1.0 | 0.8 | 0.81 |
| Respiratory complications | 50.0 | 60.7 | 0.02 |
| Cardiac complications | 9.3 | 9.0 | 0.93 |
| Acute Kidney Injury | 26.8 | 35.2 | 0.04 |
| Perioperative Stroke | 2.4 | 1.6 | 0.60 |
| Non-home discharge | 72.8 | 80.6 | 0.08 |
| Blood transfusion | 16.9 | 17.2 | 0.92 |
| **Resource utilization** |  |  |  |
| Length of stay (days) | 22 [15-34] | 18 [10-30] | 0.12 |
| Cost ($1,000) | 84 [53-137] | 72 [38-124] | 0.71 |

**Supplementary Table 3C**: Of patients with neck operations, unadjusted outcomes are compared between AWS and non-AWS. Categorial and continuous variables are reported with proportions (%) and median with interquartile range, respectively.

|  | ***Non-AWS*** | ***AWS*** | ***P-Value*** |
| --- | --- | --- | --- |
| **Clinical outcomes** |  |  |  |
| In-hospital mortality | 3.1 | 6.2 | 0.01 |
| Infectious complications | 11.1 | 23.5 | <0.001 |
| Intraoperative complications | 0.5 | 1.8 | 0.01 |
| Respiratory complications | 27.1 | 37.6 | <0.001 |
| Cardiac complications | 3.5 | 2.7 | 0.48 |
| Acute Kidney Injury | 18.5 | 20.4 | 0.48 |
| Perioperative Stroke | 0.5 | 0.4 | 0.89 |
| Non-home discharge | 40.1 | 52.2 | <0.001 |
| Blood transfusion | 14.8 | 16.4 | 0.51 |
| **Resource utilization** |  |  |  |
| Length of stay (days) | 10 [6-16] | 14 [9-22] | <0.001 |
| Cost ($1,000) | 38 [24-62] | 59 [36-86] | <0.001 |

**Supplementary Table 3D**: Of patients with thoracic operations, unadjusted outcomes are compared between AWS and non-AWS. Categorial and continuous variables are reported with proportions (%) and median with interquartile range, respectively.

|  | ***Non-AWS*** | ***AWS*** | ***P-Value*** |
| --- | --- | --- | --- |
| **Clinical outcomes** |  |  |  |
| In-hospital mortality | 4.8 | 5.8 | 0.59 |
| Infectious complications | 7.9 | 11.5 | 0.13 |
| Intraoperative complications | 0.9 | 3.6 | 0.01 |
| Respiratory complications | 14.8 | 18.0 | 0.28 |
| Cardiac complications | 26.8 | 37.4 | 0.01 |
| Acute Kidney Injury | 31.0 | 24.0 | 0.07 |
| Perioperative Stroke | 1.2 | 1.4 | 0.83 |
| Non-home discharge | 48.9 | 52.7 | 0.43 |
| Blood transfusion | 9.6 | 10.1 | 0.86 |
| **Resource utilization** |  |  |  |
| Length of stay (days) | 7 [4-11] | 10 [7-17] | <0.001 |
| Cost ($1,000) | 37 [25-57] | 48 [32-78] | 0.01 |

**Supplementary Table 3E**: Of patients with cardiac operations, unadjusted outcomes are compared between AWS and non-AWS. Categorial and continuous variables are reported with proportions (%) and median with interquartile range, respectively.

|  | ***Non-AWS*** | ***AWS*** | ***P-Value*** |
| --- | --- | --- | --- |
| **Clinical outcomes** |  |  |  |
| In-hospital mortality | 4.5 | 6.0 | 0.15 |
| Infectious complications | 14.7 | 26.6 | <0.001 |
| Intraoperative complications | 1.1 | 0.5 | 0.17 |
| Respiratory complications | 17.4 | 30.0 | <0.001 |
| Cardiac complications | 2.7 | 3.4 | <0.001 |
| Acute Kidney Injury | 19.4 | 26.0 | <0.001 |
| Perioperative Stroke | 0.3 | 0.5 | 0.65 |
| Non-home discharge | 33.7 | 44.9 | <0.001 |
| Blood transfusion | 18.9 | 22.5 | 0.06 |
| **Resource utilization** |  |  |  |
| Length of stay (days) | 8 [5-14] | 13 [7-20] | <0.001 |
| Cost ($1,000) | 30 [18-55] | 43 [25-75] | <0.001 |

**Supplementary Table 3F**: Of patients with gastrointestinal operations, unadjusted outcomes are compared between AWS and non-AWS. Categorial and continuous variables are reported with proportions (%) and median with interquartile range, respectively.

|  | ***Non-AWS*** | ***AWS*** | ***P-Value*** |
| --- | --- | --- | --- |
| **Clinical outcomes** |  |  |  |
| In-hospital mortality | 2.3 | 3.6 | <0.001 |
| Infectious complications | 18.0 | 23.8 | 0.17 |
| Intraoperative complications | 0.9 | 1.2 | 0.80 |
| Respiratory complications | 11.9 | 22.6 | 0.01 |
| Cardiac complications | 1.7 | 2.3 | 0.65 |
| Acute Kidney Injury | 30.6 | 39.3 | 0.09 |
| Perioperative Stroke | 0.5 | 0 | 0.53 |
| Non-home discharge | 40.0 | 43.2 | 0.56 |
| Blood transfusion | 15.1 | 10.7 | 0.26 |
| **Resource utilization** |  |  |  |
| Length of stay (days) | 7 [4-12] | 12 [7-17] | 0.01 |
| Cost ($1,000) | 23 [13-43] | 39 [21-71] | 0.03 |

**Supplementary Table 3G**: Of patients with genitourinary operations, unadjusted outcomes are compared between AWS and non-AWS. Categorial and continuous variables are reported with proportions (%) and median with interquartile range, respectively.

|  | ***Non-AWS*** | ***AWS*** | ***P-Value*** |
| --- | --- | --- | --- |
| **Clinical outcomes** |  |  |  |
| In-hospital mortality | 4.0 | 6.1 | 0.15 |
| Infectious complications | 10.2 | 18.2 | <0.001 |
| Intraoperative complications | 1.4 | 1.0 | 0.65 |
| Respiratory complications | 17.4 | 29.3 | <0.001 |
| Cardiac complications | 5.3 | 7.6 | 0.15 |
| Acute Kidney Injury | 22.1 | 24.7 | 0.38 |
| Perioperative Stroke | 1.2 | 0.5 | 0.35 |
| Non-home discharge | 57.3 | 65.0 | <0.001 |
| Blood transfusion | 23.6 | 22.7 | <0.001 |
| **Resource utilization** |  |  |  |
| Length of stay (days) | 9 [5-17] | 13 [8-21] | 0.01 |
| Cost ($1,000) | 39 [22-71] | 52 [29-102] | 0.02 |

**Supplementary Table 3H**: Of patients with vascular operations, unadjusted outcomes are compared between AWS and non-AWS. Categorial and continuous variables are reported with proportions (%) and median with interquartile range, respectively.
